# Long‐lasting visceral hypersensitivity in a model of DSS‐induced colitis in rats

**DOI:** 10.1111/nmo.14441

**Published:** 2022-08-05

**Authors:** Sergio López‐Estévez, Josep Manuel López‐Torrellardona, Marc Parera, Vicente Martínez

**Affiliations:** ^1^ Department of Cell Biology, Physiology and Immunology Universitat Autònoma de Barcelona Barcelona Spain; ^2^ Neuroscience Institute Universitat Autònoma de Barcelona Barcelona Spain; ^3^ Centro de Investigación Biomédica en Red de Enfermedades Hepáticas y Digestivas (CIBERehd) Instituto de Salud Carlos III Madrid Spain

**Keywords:** colitis, hypersensitivity, intestinal inflammation, pain sensitization, visceral pain

## Abstract

**Background:**

Persistent visceral hypersensitivity is a key component of functional and inflammatory gastrointestinal diseases. Current animal models fail to fully reproduce the characteristics of visceral pain in humans, particularly as it relates to persistent hypersensitivity. This work explores the validity of DSS‐induced colitis in rats as a model to mimic chronic intestinal hypersensitivity.

**Methods:**

Exposure to DSS (5% for 7 days) was used to induce colitis in rats. Thereafter, changes in viscerosensitivity (visceromotor responses to colorectal distension—CRD), the presence of somatic referred pain (mechanosensitivity of the hind paws, von Frey test) and the expression (qRT‐PCR) of sensory‐related markers (colon, lumbosacral DRGs, and lumbosacral spinal cord) were assessed at different times during the 35 days period after colitis induction.

**Results:**

Following colitis, a sustained increase in visceromotor responses to CRD were observed, indicative of the presence of visceral hypersensitivity. Responses in animals without colitis remained stable over time. In colitic animals, somatic referred hypersensitivity was also detected. DSS‐induced colitis was associated to a differential expression of sensory‐related markers (with both pro‐ and anti‐nociceptive action) in the colon, lumbosacral DRGs and lumbosacral spinal cord; indicating the presence of peripheral and central sensitization.

**Conclusions and Inferences:**

DSS‐induced colitis in rats is associated to the generation of a long‐lasting state of visceral (colonic) hypersensitivity, despite clinical colitis resolution. This model reproduces the changes in intestinal sensitivity characteristics of inflammatory and functional gastrointestinal disorders in humans and can be used in the characterization of new pharmacological treatments against visceral pain.

**Highlights**

- Rats with DSS‐induced colitis develop long‐lasting visceral hypersensitivity, as assessed during colorectal distension.
- Intestinal hypersensitivity is likely to result from the interaction between central and peripheral sensitizing mechanisms.
- This model represents a valid tool for the characterization of new therapeutic targets and the validation of new drugs for the treatment of visceral pain of intestinal origin.

## 1 INTRODUCTION

Visceral hypersensitivity is a common component of inflammatory and functional gastrointestinal (GI) disorders in humans. Indeed, persistent alterations in GI sensitivity with altered pain thresholds and signs of hypersensitivity are considered as key findings in functional GI disorders, particularly in irritable bowel syndrome (IBS).^1^, ^2^ Similar long‐lasting alterations in sensitivity, although with some inconsistencies, are also reported during the active and inactive phases of inflammatory bowel disease (IBD).^3^, ^4^ Despite the clinical importance of visceral pain, the development of effective treatments directed towards the specific control of this pain modality has been hampered by: (i) the complex physiology of visceral pain; (ii) the differences in the neural and immune substrata underlying visceral vs. somatic pain; and (iii) the difficulties in developing animal models with high translational validity, particularly as it relates to persistent/chronic hypersensitivity.^5^, ^6^, ^7^

Taking this into account, numerous animal models have been developed for the assessment of visceral sensitivity in normal conditions or in states of sensitization. A systematic review of these models is out of the scope of the present work and can be found elsewhere [see as examples: Regmi and Shah (2020); Johnson et al. (2020); West and McVey Neufeld (2021); Accarie and Vanuytsel (2020)].^8^, ^9^, ^10^, ^11^ Several animal models of intestinal hypersensitivity have been developed based on the evidence that inflammation seems to be a pathophysiological component of inflammatory and functional GI disorders, as mentioned above. Overall, postinflammatory models have been based on the local administration (intracolonic enemas) of different active compounds (such as acetic acid, capsaicin, mustard oil, zymosan, trinitrobenzene sulfonic acid (TNBS)‐ or dinitrobenzene sulfonic acid (DNBS))^12^, ^13^, ^14^, ^15^, ^16^ or the experimental infection with biological agents (such as *Trichinella spiralis*, *Nippostrongylus brasiliensis* or *Campylobacter* species).^17^, ^18^, ^19^ From the available models, a recent report recommends the use of the intracolonic TNBS postinflammatory model of colonic hypersensitivity as the principal model for screening novel treatments for visceral pain originating within the GI tract.^9^ This recommendation was based on four characteristics of the model: simplicity, characterization in rodent species, reproducibility across laboratories and construct validity and translational relevance. However, data available on the TNBS model is limited and can be considered insufficient as to endorse its use over similar models.

One of the most commonly used models of intestinal inflammation is based on the dextran sulfate sodium (DSS) exposure in rodents. This model has strong similarities with the characteristic histopathology of intestinal inflammation in humans and it is regarded as a valid model of human intestinal inflammation. The DSS‐induced colitis model has been extensively validated in both rats^20^, ^21^, ^22^, ^23^, ^24^, ^25^, ^26^ and mice.^7^, ^27^, ^28^, ^29^ Some reports suggest that the DSS model elicits an inflammatory response more similar to human IBD than the TNBS model.^30^ In fact, exposure to DSS elicits a colitic response that, according to its histopathological features and immunologic profile, resembles human ulcerative colitis, while TNBS‐induced colitis is more similar to Crohn's disease.^31^ Visceral pain responses during DSS‐induced colitis have been studied mainly in mice. Although with some contradictory data, several studies consistently indicate the presence of intestinal (colonic) hypersensitivity during DSS‐induced inflammation, either acute or chronic.^32^, ^33^, ^34^, ^35^, ^36^

With the objective of broadening the available models to assess visceral pain, particularly as it related to the presence of chronic hypersensitivity, the present work evaluates the validity of DSS‐induced colitis in rats as a model to assess intestinal (colonic) sensitivity, with special emphasis in the presence of long‐term sensitization. Moreover, we also assessed changes in the expression of sensory‐related markers at the periphery (colon and dorsal root ganglion) or the spinal cord level, as a way to gain insight into the mechanisms underlying inflammation‐associated changes in viscerosensitivity following DSS exposure.

## 2 MATERIALS AND METHODS

### 2.1 Animals

Female adult Sprague–Dawley rats (Crl:OFA[SD]; 10–12 week–old at the beginning of the study; Charles River, France) were used. Female rats were used because of the higher prevalence of GI dysfunctions associated with visceral pain, such as IBS, in women.^37^, ^38^ Given the inconsistent observations related to the potential effect of the estrous cycle in sensitivity, the phase of the cycle was not taken into consideration in the present studies.^39^, ^40^, ^41^, ^42^, ^43^, ^44^ Animals were group‐housed (3–4 animals per cage) in standard cages and were maintained in conventional conditions in an environmentally controlled room (20–22°C, 12 h light: dark cycle), with food and water ad libitum, except when receiving DSS. Animals were allowed to acclimatize to the animal facility for at least 1 week prior to the start of the studies. All experiments were performed in accordance with EU regulations and were approved by the Ethical Committee of the Universitat Autònoma de Barcelona (protocols 3958 and 3961) and the Generalitat de Catalunya (protocol 9915).

### 2.2 DSS‐induced colitis

Rats received a solution of 5% DSS (45 kDa; TdB Consultancy AB, Upssala, Sweden) in their drinking water for 7 days to induce colitis. The same protocol has been previously used for the induction of colitis in rats.^20^, ^21^, ^22^, ^23^, ^24^ DSS solutions were prepared freshly on a daily basis while control rats received normal tap water. Throughout the study, body weight, the general state, and the presence of clinical signs of inflammation were assessed individually on a daily basis.

### 2.3 Assessment of colonic sensitivity: colorectal distension

For the assessment of colonic sensitivity, the colorectal distension (CRD) method was used, following, with minor modifications, previously published procedures.^45^ Before starting experiments, rats were gradually habituated (45–75 min per day, at 15 min steps) to Bollmann cages to reduce motion artifacts and minimize the effects of stress‐related responses. On the day of the experiment, rats were anesthetized with isoflurane (Isoflo; Esteve, Barcelona, Spain). A plastic balloon (made in‐house; length: 2.5 cm; diameter: 1.5 cm) with connecting catheter was inserted in the distal colon (2 cm from the base of the balloon to the anus). The catheter was fixed with tape to the tail of the animal. Animals were then placed in Bollmann cages and allowed to recover from anesthesia for at least 20–30 min before starting the CRD protocol.

The balloons were connected to pressure transducers (P‐602, CFM‐k33, 100 mmHg, Bronkhorst HI‐TEC, Veenendal, The Netherlands) to control intraballoon pressure during the CRD procedure. A customized barostat system was used to manage balloon inflation and to measure pressure changes during CRD. The pressure was monitored and kept constant by a custom computer software (Pharmlab 5.0 online, AstraZeneca R&D, Mölndal, Sweden) running on a standard computer. The same system served also for data acquisition.

For CRD, a protocol consisting of repetitive phasic distensions at 80 mmHg, with a pulse duration of 30 s at 5 min intervals was used. This protocol served to induce acute mechanical hypersensitivity and has been extensively used to assess colonic mechanosensitvity.^45^, ^46^, ^47^, ^48^ Rapid pressure changes in the balloon, which reflect contractions of the abdominal muscles, served to assess visceromotor responses (VMRs). The analogue input channels were sampled with individual sampling rates, and digital filtering was performed on the signals. The balloon pressure signals were sampled at 50 Hz. A highpass filter at 1 Hz was used to separate the contraction‐induced pressure changes from the slow varying pressure generated by the barostat. A customized software (CDR Analytics v2.0, J4 Style) was used to quantify the magnitude of the balloon pressure signals. The average rectified value of the balloon pressure signals was calculated for 30 s before the pulse (i.e., baseline activity) and for the duration of the pulse. This measure allows the contractions of the abdominal muscles to be distinguished from the relaxations or compensations of the barostat. When calculating the magnitude of the balloon pressure signals, the first and last 4.5 s of each pulse were excluded, since these reflect artifacts produced by the barostat during the inflation and deflation process and do not originate from the animal. The total response to a CRD protocol was calculated as the area under curve (AUC) of the corresponding VMRs registered during the distension time, corrected for the basal activity.

### 2.4 Evaluation of referred mechanical somatic hyperalgesia: von Frey test

To evaluate referred mechanical sensitivity, animals were located into compartment enclosures in a test chamber with a framed metal mesh floor, through which von Frey monofilaments were applied for pain assessment (bending force range from 1 to 26 g; North Coast Medical, Inc.; Gilroy, California, USA). Animals were habituated to the testing environment for a 30–40 min period before starting the procedure. Somatic referred pain was quantified by measuring the withdrawal response to punctate mechanical stimulation of the hind paws, as previously described.^7^ For each animal, the mean measure of the right and left hid paw was taken as the measure of sensitivity. The up‐down method was used to assess pain thresholds, which represent the mechanical threshold that elicits 50% of the maximal response.^49^ The percentage of DSS‐induced algesic effect was calculated as: % = [(PWB ‐ PWI) / PWB] * 100, where PWB and PWI are the basal paw threshold (g) and paw threshold on the day of interest, respectively.

### 2.5 Experimental protocols

**FIGURE 1:**
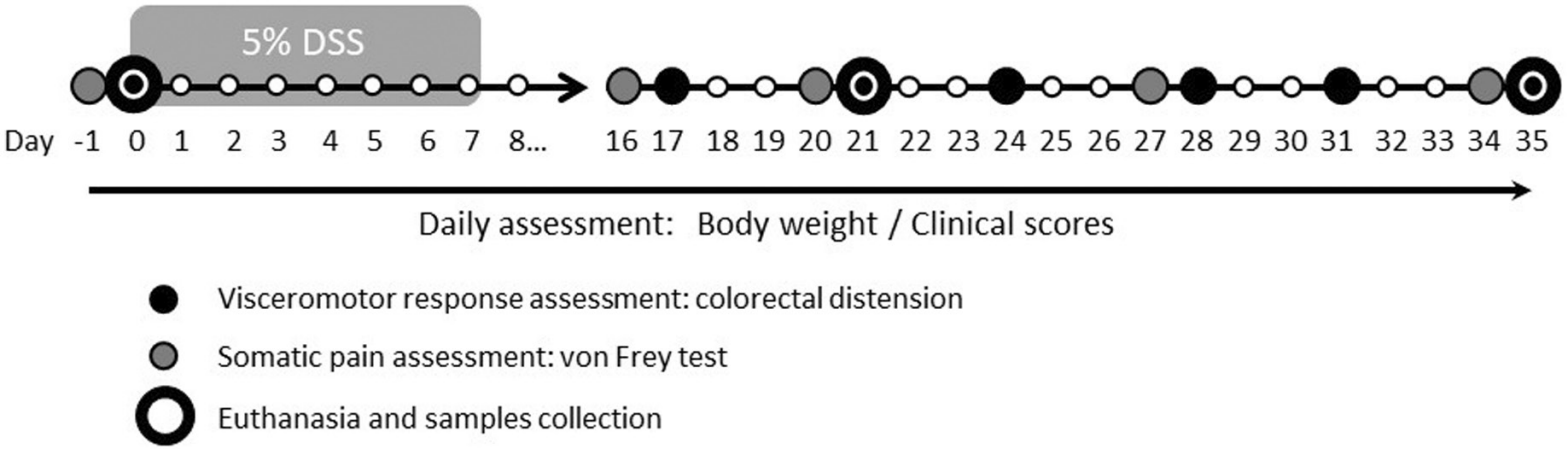
Schematic representation of the experimental protocols followed in the study

Animals were weighted and randomly distributed in two experimental groups (control and colitis; experimental Day −1). Thereafter they received 5% DSS during 7 consecutive days to induce colitis (experimental Days 0–7). The control group received normal tap water during the same period. After finishing the induction of colitis, a resting period of 9 days (experimental Days 7–16) was allowed and thereafter somatic (von Frey test) and visceral sensitivity was assessed at regular intervals throughout the following 20 days period (experimental Days 16–35), with a 3–4 days between consecutive tests. Basal somatic and visceral sensitivity were assessed at the start of the DSS exposure (experimental Days −1 and 0 for somatic and visceral sensitivity, respectively). In addition, at experimental Days 0 (basal), 21, and 35, some animals were euthanized for the assessment of inflammation (see below). Figure 1 shows a schematic representation of the experimental protocols.

### 2.6 Samples collection

At experimental Days 0, 21, and 35, control and DSS‐treated rats were deeply anesthetized with isoflurane (Isoflo; Esteve, Barcelona, Spain) and euthanatized by exsanguination through intracardiac puncture. Thereafter, a medial laparotomy was performed, the ceco‐colonic region localized and the colon dissected. Afterward, a tissue sample from the proximal colon was collected and frozen immediately in liquid nitrogen. In addition, dorsal root ganglia (DRGs) corresponding to the lumbosacral region and the lumbosacral region of the spinal cord (L3‐S2) were also collected and frozen immediately. Frozen samples were stored at −80°C until analysis. Moreover, tissue samples (about 3 cm) of the middle colon were collected and fixed overnight in 4% paraformaldehyde. Afterward, tissues were paraffin embedded and 5 μm‐thick sections obtained.

### 2.7 Clinical and macroscopic assessment of inflammation

Clinical assessment of inflammation included daily monitoring of body weight, appearance of feces, and general health condition, as previously described.^7^, ^28^ A score (0–8) was assigned for health condition (including hunch posture, piloerection, fecal consistency, and anal inflammation); where 0 indicates normal activity/fur/fecal content/no anal inflammation, 1 indicates abnormal gait/bristly fur/wet anus/loose fecal content and 2 indicates prostrated animal/dirty fur/watery or bloody anus/diarrhea.

At necropsy, the macroscopic appearance of the colon (inflammatory score) was scored based on: consistency of fecal content (score 0–3) and presence of visible fecal blood (score 0–3). Additionally, the extent of oedema (0–3), stiffness (0–2), thickness (0–3), and presence of ulcerations (0–1) were also assessed; equivalent to a maximum total score of 15.

### 2.8 Histopathological studies

Hematoxylin–eosin‐stained sections were obtained from 4% paraformaldehyde‐fixed colon specimens, following standard procedures, for their histological examination. A histopathological score (ranging from 0, normal, to 12, maximal alterations) was assigned to each animal. Parameters scored included: structure of the luminal epithelium (0: normal; 1: mild alterations; 2: local destruction; 3: generalized destruction), state of the lower part of the crypts (0: normal; 1: mild alterations; 2: local destruction; 3: generalized destruction), occurrence of edema (0: normal; 1: mild local edema in submucosa and/or lamina propria; 2: moderate diffuse edema in submucosa and/or lamina propria; 3: severe generalized edema in submucosa and/or lamina propria), and presence of inflammatory infiltrate (0: normal; 1: mild localized infiltrate; 2: mild generalized infiltrate; 3: severe generalized infiltrate). Histopathological scoring was performed in a blinded manner (coded slides) by two independent researchers.

### 2.9 Gene expression: quantitative reverse transcription‐PCR

Total RNA was extracted from frozen tissue samples using TRI reagent with Ribopure Kit (Ambion/Applied biosystems, Foster City, California, USA). RNA was purified by precipitation with lithium chloride.^50^ Thereafter, a two‐step quantitative real‐time PCR (RT‐qPCR) was performed. RNA samples were converted into cDNA with a High‐Capacity cDNA Reverse Transcription Kit (Applied Biosystems). Real‐time quantitative PCR (iTaq Universal™ SYBR® Green Supermix, Bio‐Rad) was incubated on the Bio‐Rad CFX384 Touch Real‐Time PCR Detection System (Bio‐Rad). All samples were analyzed in triplicate. For each sample, the cycle thresholds were obtained and data were analyzed using the comparative Ct method (2^−ΔΔCt^), non‐inflammed vehicle‐treated animals served as the calibrator.^51^ PrimePCR™ SYBR® Green Assay (Bio‐Rad) for interferon γ (IFN‐γ; qRnoCID0006848), interleukin 1β (IL‐1β; qRnoCID0004680), and interleukin 10 (IL‐10; qRnoCID0005930) were used for inflammatory response. Cannabinoid receptor type 1 (CB1; qRnoCED0008430) and type 2 (CB2; qRnoCED0008595), μ‐opioid receptor (MOR; qRnoCED0003071), transient receptor potential vanilloid 1 (TRPV1; qRnoCID0003147), and 3 (TRPV3; qRnoCID0007727), nerve growth factor (NGF; qRnoCID0003911), receptor activity modifying protein 1 (Ramp1; qRnoCED0001653), and σ1 receptor (σ1R; qRnoCED0005919) were used for the assessment of neuronal/sensory activity. Actin β (Actb; qRnoCID0056984) and hypoxanthine‐guanine phosphoribosyltransferase 1 (Hprt1; qRnoCED0057020) were used as endogenous reference genes.

### 2.10 Statistical analysis

Data are expressed as mean ± SEM. For RT‐qPCR data, a robust analysis (one iteration) was used to obtain mean ± SEM. Data were analyzed by one‐ or two‐way ANOVA, with or without repeated measures, as appropriate, followed, when necessary, by a Bonferroni's multiple comparisons test. Data were considered statistically significant when *p* < 0.05. All statistical analyses were performed using SPSS program (version 17 for Windows, IBM, Madrid, Spain) or GraphPad Prism 7 (GraphPad Software, La Jolla, California, USA).

## 3 RESULTS

In non‐inflamed animals, none of the parameters assessed showed time‐related changes. Therefore, control data obtained at experimental Days 0, 21, and 35 have been pooled as a single control group.

### 3.1 Colitis development and follow‐up

**FIGURE 2:**
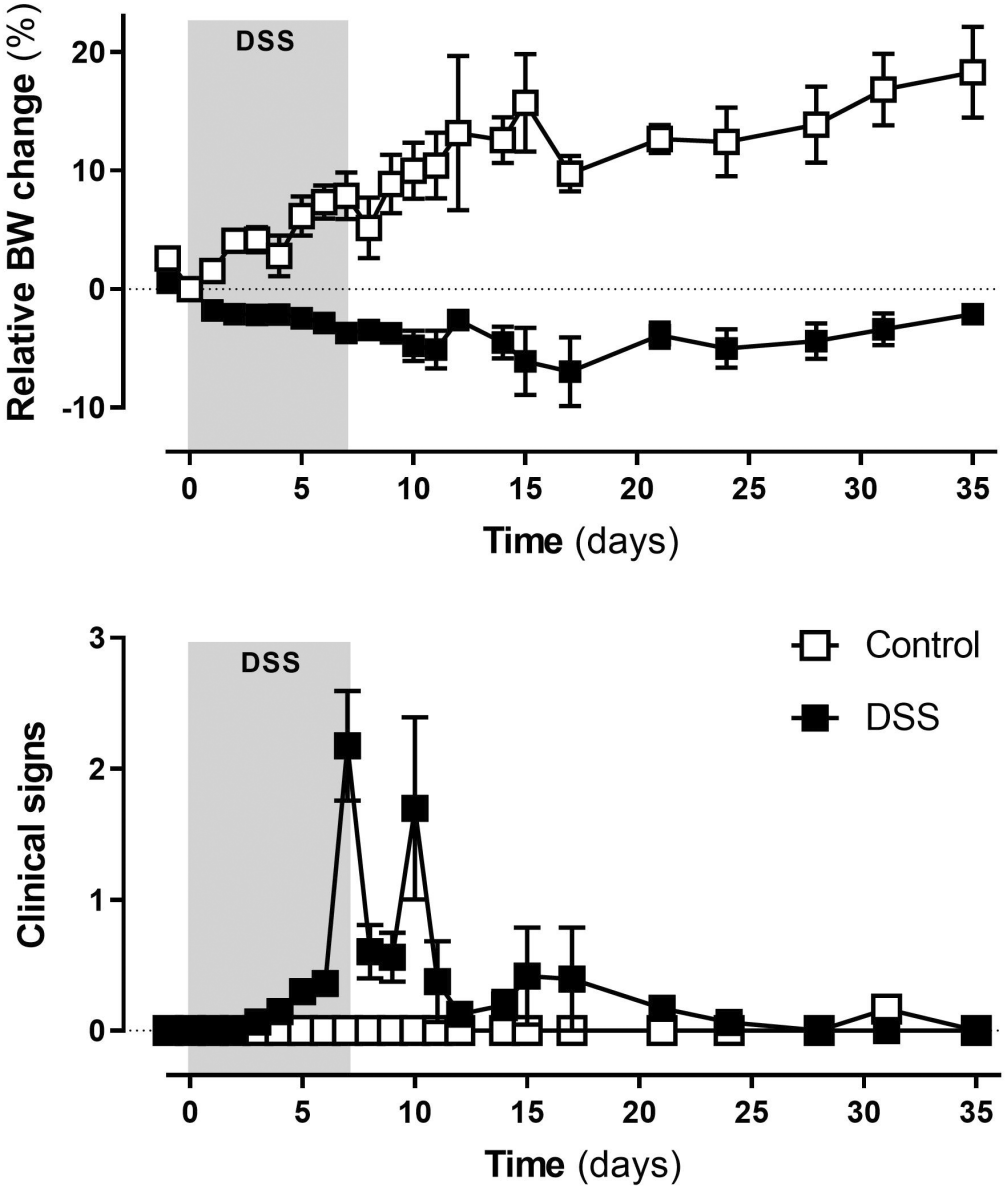
Clinical characterization of DSS‐induced colitis in rats: time‐course changes in body weight (% change from Day 0, taken as 100%) and clinical signs. The gray square denotes the period of DSS exposure. Data are mean ± SEM (*n* = 5–16)

Rats not exposed to DSS showed a linear body weight increase throughout the experimental time, with no clinical signs of inflammation. In contrast, animals receiving DSS showed changes in body weight and presented clinical signs, consistent with the development of colitis, in a time‐related manner (Figure 2). Body weight showed a decline from experimental Day 2 (*p* < 0.05 vs. body weight at Day 0; *p* < 0.05 vs. body weight in animals without colitis at the corresponding days), with a peak reduction of 7.0 ± 2.9% by Day 17. Thereafter, body weight increased slowly but without recovering the growth ratio observed in control conditions (Figure 2A). In the same animals, clinical signs of colitis, such as piloerection, loose feces/watery diarrhea, and fecal blood were observed, with a peak between experimental Days 7 and 10 (*p* < 0.05 vs. control group; Figure 2B), likely reflecting individual variability in the response to DSS and the development of inflammation. Thereafter, clinical signs subsided and were absent from experimental Day 21 onward.

**FIGURE 3:**
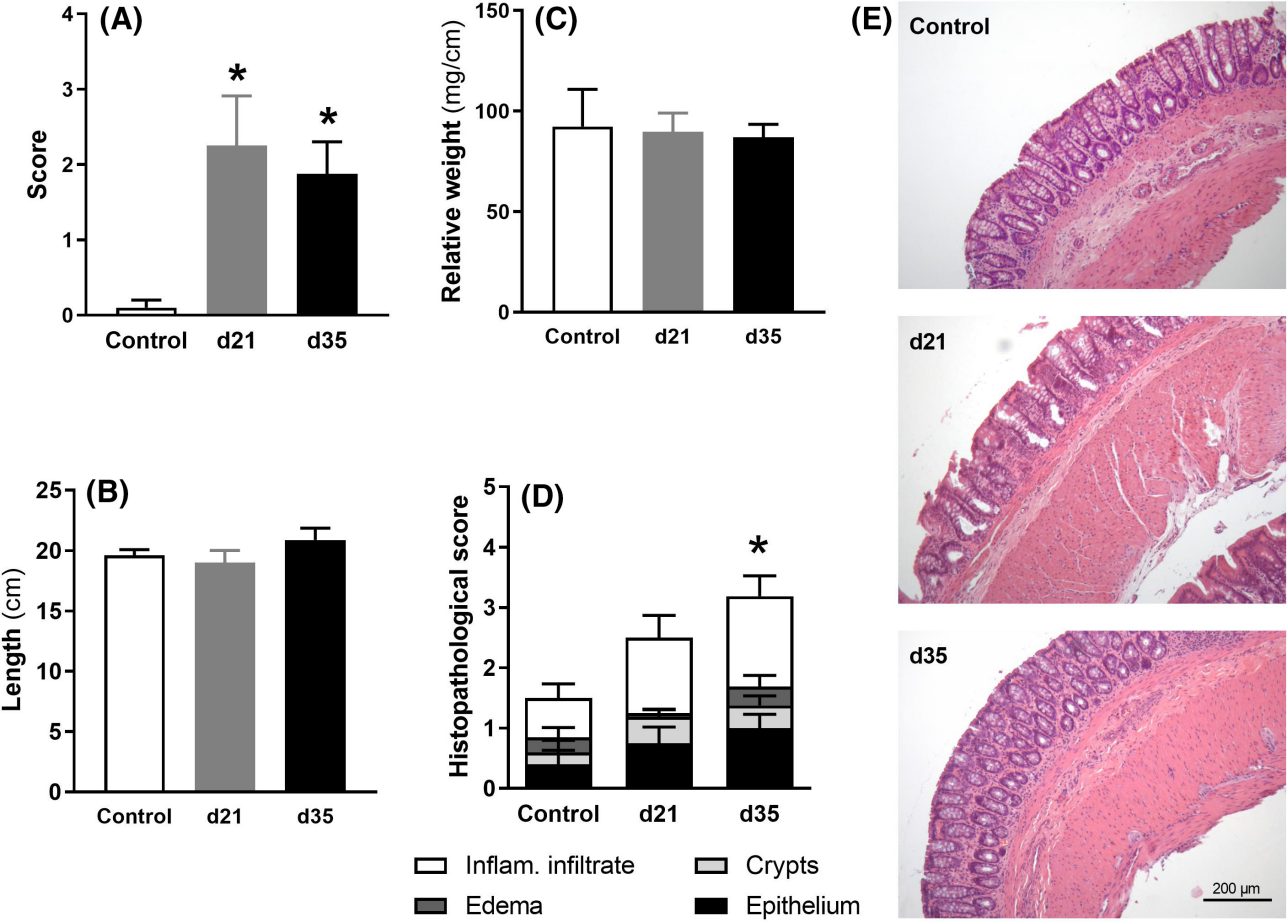
Macro‐ and micro‐scopic assessment of the colon at the time of necropsy in healthy controls and animals with DSS‐induced colitis at experimental Days 21 (d21) and 35 (d35). A: macroscopic assessment of the colon (maximum possible scoring: 15; see methods for details); B: colon length; C: colonic relative weight; D: colonic histopathological scores (maximum possible scoring: 12; see methods for details). Data are mean ± SEM of 4–5 animals per group. *: *p* < 0.05 vs. control group (in panel D, used for the complete histopathological score). E: Representative microphotographs showing hematoxylin–eosin‐stained colonic sections from control and DSS‐treated animals at experimental Days 21 (d21) and 35 (d35)

At experimental Days 21 and 35, the colon of animals exposed to DSS showed some macroscopic signs of inflammation (Figure 3A–C) and histopathological alterations, particularly as it relates to the presence of inflammatory infiltrate through the colonic wall (Figure 3D‐E).

**FIGURE 4:**
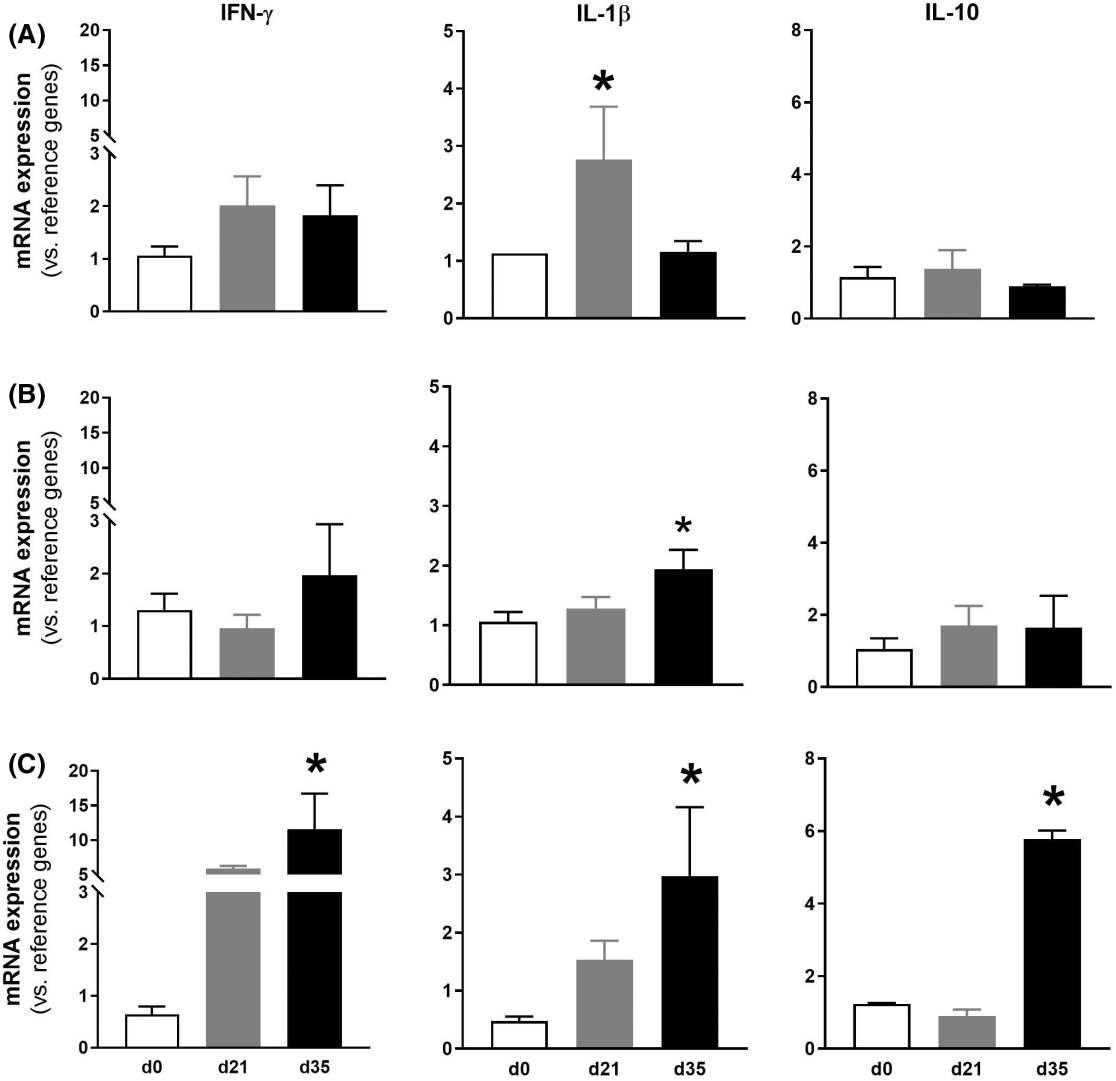
Effects of DSS‐induced colitis on the expression of pro‐ (IFN‐γ and IL‐1β) and anti‐inflammatory cytokines (IL‐10) in colon (A), lumbosacral DRGs (B), and lumbosacral spinal cord (C) in control conditions (denoted at experimental Day 0, d0) and at experimental Days 21 (d21) and 35 (d35). Data are mean ± SEM, *n* = 4–5 per group. *: *p* < 0.05 vs. control group

In all cases, expression (RT‐qPCR) of IFN‐γ, IL‐1β, and IL‐10 was detectable and quantifiable in colon, lumbosacral DRGs, and lumbosacral spinal cord. In animals with colitis, time‐related changes in the expression of colonic proinflammatory cytokines, IFN‐γ and IL‐1β, with an up‐regulation by experimental Day 21 (IL‐1β: *p* < 0.05 vs. control; Figure 4A) and a normalization in the expression by Day 35 were observed. Similar changes, although delayed in time, were detected in DRGs and spinal cord. In this case, up‐regulation of pro‐inflammatory cytokines increased with time, reaching statistically significant values at experimental Day 35 (Figure 4B,C).

The gene expression of the anti‐inflammatory cytokine IL‐10 was only modified at the lumbosacral spinal cord at experimental Day 35, showing a significant up‐regulation (*p* < 0.05 vs. control; Figure 4).

### 3.2 Effects of DSS‐induced colitis on colonic sensitivity

**FIGURE 5:**
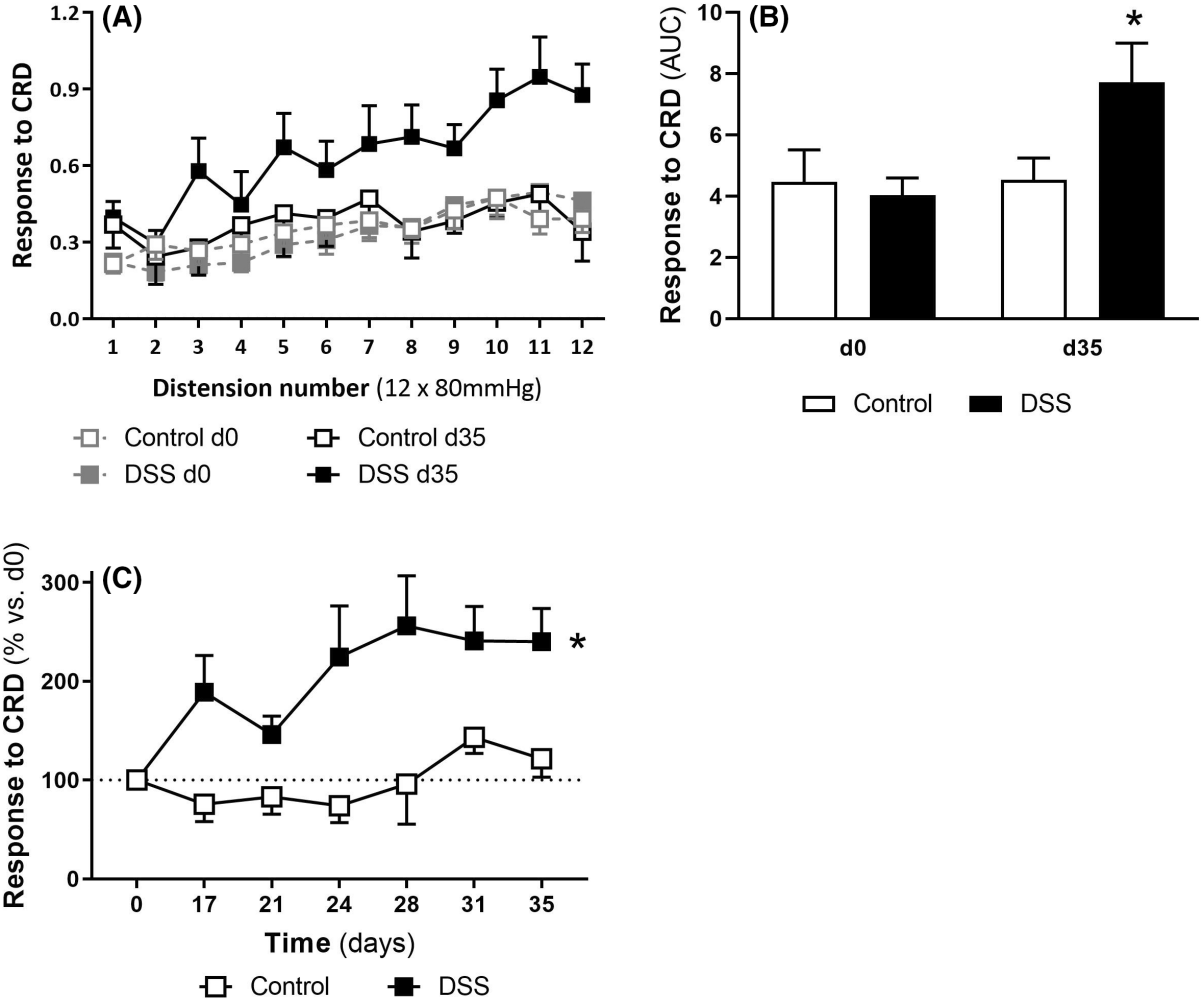
Effect of DSS‐induced colitis on VMR responses to phasic repetitive CRD. A: VMRs to repetitive phasic CRD in healthy and DSS‐induced colitic rats. VMRs to CRD were assessed in the same animals, either healthy or colitic, before the induction of colitis, experimental Days 0 (d0), and 35 days after DSS exposure (d35). B: Cumulative responses to phasic CRD (AUC) of the experimental groups included in A. C: Time‐course changes in the cumulative responses to phasic CRD (AUC) throughout the experimental time. Data represents % change from the VMRs determined in basal conditions at experimental Day 0, taken as 100%. Data are mean ± SEM (*n* = 7–16). *: *p* < 0.05 vs. control group (two‐way ANOVA with time and inflammation as factors)

In control conditions (experimental Day 0), repetitive phasic CRD at 80 mmHg elicited VMRs similar to those described elsewhere when using this protocol. CRD evoked a significant increase in VMRs vs. basal activity (first distension: 0.21 ± 0.04; *p* < 0.05 vs. basal activity). Moreover, VMRs showed a distension‐related increase along the CRD protocol (first distension: 0.21 ± 0.04, *p* < 0.05 vs. 12th distension: 0.39 ± 0.5), with an overall 201 ± 27% increase in the response observed from the first to the last distension (Figure 5A). Animals not exposed to DSS maintained this response to phasic CRD throughout the experimental time with only small, non‐significant, fluctuations, as assessed in experimental Days 17, 21, 24, 28, 31, and 35 (*p* > 0.05 two‐way ANOVA with time and inflammation as factors; Figure 5). Animals exposed to DSS showed increased VMRs to phasic CRD from experimental Day 17 when compared with healthy animals. Increased responses to CRD were maintained on time until the end of the experimental time (*p* < 0.05 vs. control; Figure 5C). As an example, at experimental Day 35, in colitic animals VMRs to phasic CRD increased from the first (0.40 ± 0.06) to the last distension (0.88 ± 0.12; *p* < 0.05 vs. VMR during the first distension), with an overall 255 ± 33% increase from the first to the last distension (Figure 5).

### 3.3 Effects of DSS‐induced colitis on referred somatic mechanical sensitivity

**FIGURE 6:**
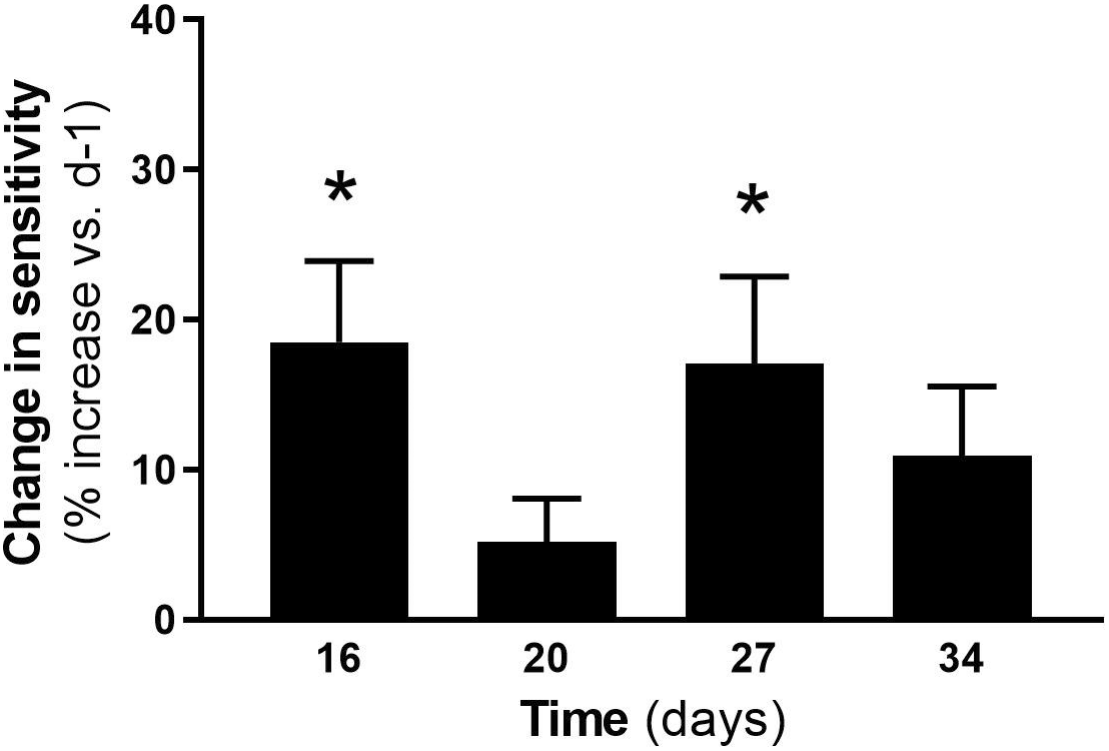
Time course changes in mechanical referred somatic sensitivity in animals with DSS‐induced colitis as assessed in the hind paws [relative to experimental Day −1 (d‐1), taken as basal sensitivity, 0% change] at experimental Days 16 (d16), 20 (d20), 27 (d27) and 34 (d34) (see Figure 1 for details of the experimental protocol). Data are mean ± SEM of *n* = 8 animals per group. *: *p* < 0.05 vs. basal sensitivity (experimental Day −1)

In colitic rats, small fluctuations in referred mechanical sensitivity, manifested as a reduction in the force needed to evoke a paw withdrawal reflex, was observed from experimental Day 16 (vs. sensitivity measures determined at experimental Day −1, taken as basal sensitivity). However, significance was only achieved at experimental Days 16 and 27 (Figure 6) and no correlation with visceromotor responses during CRD was found (see Figure S1). No changes in somatic sensitivity were observed in control conditions (data not shown).

### 3.4 Effects of DSS‐induced colitis on sensory‐related markers

**FIGURE 7:**
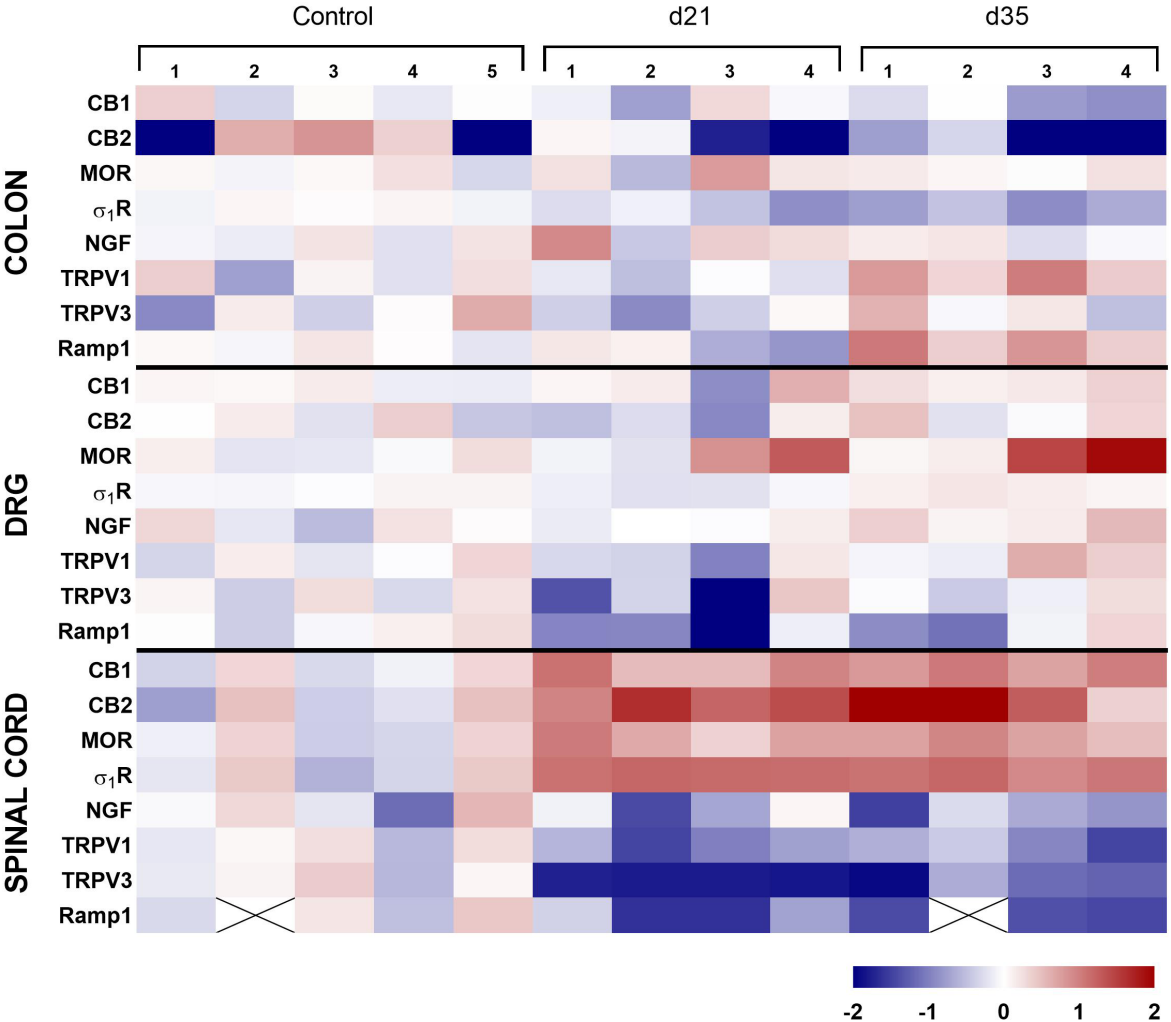
Heat map showing the relative expression (mRNA) of sensory‐related markers in colon, lumbosacral DRGs, and lumbosacral spinal cord in control conditions (healthy animals) and after DSS‐induced colitis [experimental Days 21 (d21) and 35 (d35)]. The cross (X) denotes the two spinal cord samples in which no expression of Ramp1 was detected. See also Figure 8

**FIGURE 8:**
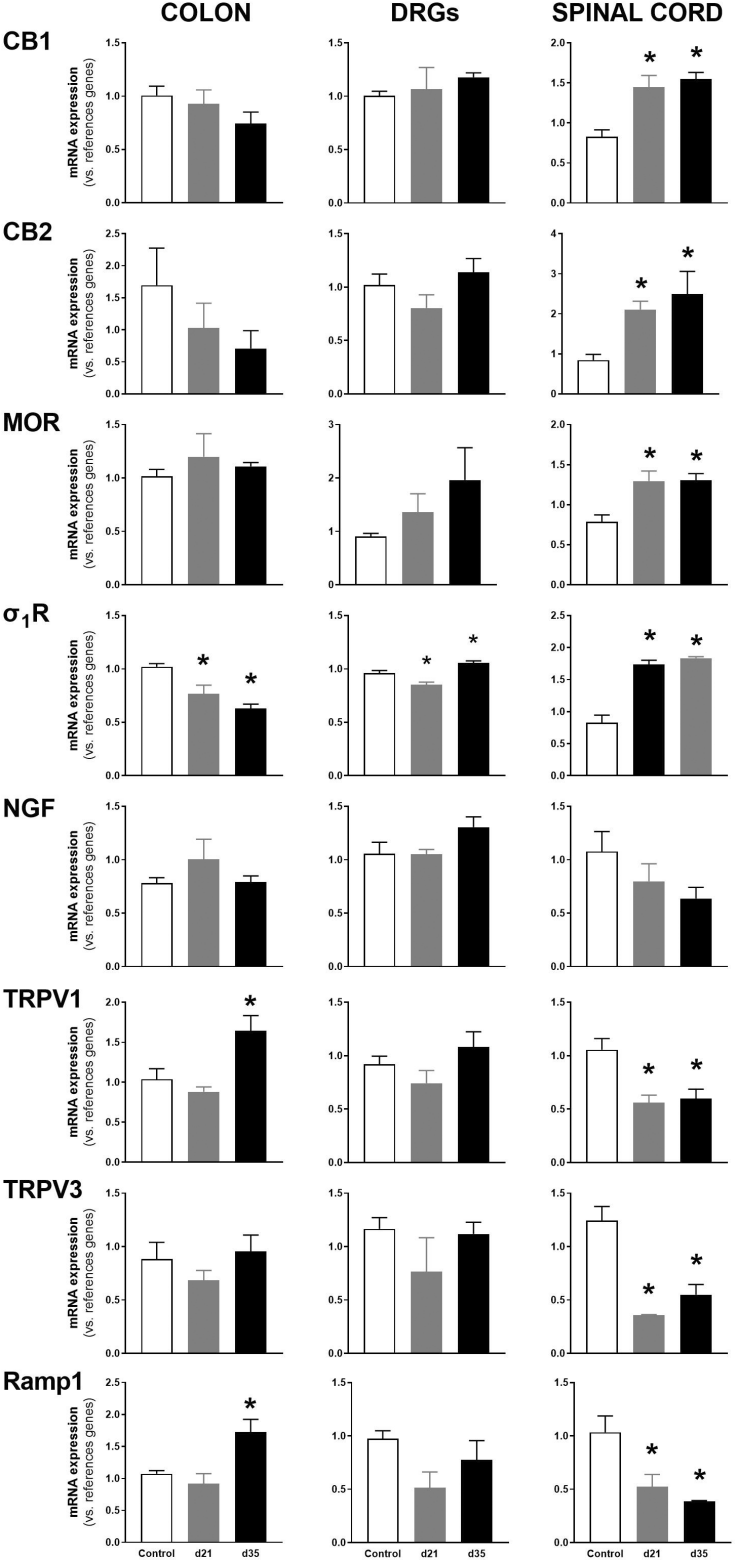
Relative expression (mRNA) of sensory‐related markers in colon, lumbosacral DRGs, and lumbosacral spinal cord in control conditions and after DSS‐induced colitis [experimental Days 21 (d21) and 35 (d35)]. Data are mean ± SEM of 4–5 animals per group (as shown individually in Figure 7). *: *p* < 0.05 vs. control

mRNA for the sensory‐related markers evaluated was detected in all samples, with the exception of Ramp1 which was not detected in two spinal cord samples. In control rats, expression levels remained stable over time. As it relates to the colon, in animals with DSS‐induced colitis, only the expression of TRPV1 and Ramp1, on experimental Day 35, was affected, with a significant up‐regulation (both *p* < 0.05 vs. control; Figures 7 and 8). On the contrary, colonic expression of σ_1_Rs was significantly down‐regulated (*p* < 0.05 vs. control, for experimental Days 21 and 35; Figures 7 and 8).

In lumbosacral DRGs, only σ_1_R expression was affected, with a dual response including a down‐regulation at early times (experimental Day 21) (*p* < 0.05 vs control) followed by an up‐regulation at experimental Day 35 (*p* < 0.05 vs. control; Figures 7 and 8).

Within the lumbosacral spinal cord, an overall up‐regulation of anti‐nociceptive‐related markers (*p* < 0.05 for CB1, CB2, and MOR vs. control; Figures 7 and 8) and a down‐regulation of pro‐nociceptive‐related markers (*p* < 0.05 for TRPV1 and 3 and Ramp1 vs. control; Figures 7 and 8) was detected following DSS‐induced colitis. Spinal σ_1_Rs were up‐regulated following DSS exposure (*p* < 0.05 vs. controls, regardless the time considered; Figures 7 and 8).

## 4 DISCUSSION

Robust models for the study of visceral pain and the characterization of potential therapeutic targets are still a need. Taking into account the clinical characteristics of visceral pain of intestinal origin these models should, ideally, involve states of long‐lasting hypersensitivity. In the present report, we present evidence supporting the potential value of DDS‐induced colitis in rats as a way to induce long‐lasting colonic hypersensitivity, thus providing an animal model to investigate visceral pain mechanisms, to characterize new therapeutic targets and for the validation/screening of new drugs.

It is well recognized that inflammation and pain are associated phenomena in multiple organic systems, including somatic and visceral structures. Within the GI tract, inflammatory and functional bowel disorders are characterized by the coexistence of inflammation, of variable intensity, and visceral pain, with chronic states of hypersensitivity as a distinctive feature.^1^, ^2^ Taking this into account, animal models to study visceral pain arising from the gut are largely based on the induction of inflammation or irritation (with the consequent immune activation) of the GI tract.^8^, ^9^, ^11^, ^52^ In this context, DSS‐induced colitis is a well‐validated and accepted model of human colitis (in particular ulcerative colitis).^7^, ^27^, ^28^, ^29^ Compared with other inflammatory models, such as TNBS/DNBS‐induced inflammation, the DSS model induces a less aggressive inflammatory response that, according to some authors, resembles in a more accurate manner the inflammation observed in humans.^30^ In the present conditions, exposure to DSS led to the induction of a state of colitis, as determined by the presence of clinical signs consistent with those previously reported for this model.^21^, ^22^, ^23^, ^24^ As previously characterized in mice, in rats DSS exposure leads to an acute state of colitis that partially resolves over time^21^, ^26^ generating a state of chronic, low‐degree, inflammation, which was evident up to 4 weeks after the induction process. During this state, although no clinical signs were noticed and a gain of body weight was observed, histopathological and molecular alterations (moderate up‐regulation of pro‐inflammatory cytokines) consistent with the presence of mild inflammation were still detected. These long‐term changes are reminiscent of the quiescent phases observed in IBD patients when the flares of the disease resolve and of the low degree of inflammation/immune activation described in IBS patients.^1^, ^2^ Therefore, the model might bear utility reproducing the functional/structural/molecular alterations of IBS or latent IBD.

Interestingly, evidence of long‐lasting immune activation outside the intestine, specifically in neural substrata important in sensory processing from the colon, that is, lumbosacral DRGs and spinal cord was also observed. Moreover, expression changes of immune‐related mediators at these locations increased over time, thus further supporting the presence of long‐lasting molecular alteration, even outside the original site of inflammation, with potential significance in the processing of afferent sensory signals. These changes might suggest an inflammation‐associated remodeling of central (spinal) mechanisms that might be related to alterations in pain sensitivity, as previously suggested for other models of gastrointestinal inflammation, such as during *T. spiralis*‐induced enteritis.^53^ Altogether, these observations support the presence of changes in visceral sensitivity after clinical DSS‐induced colitis resolution.

In animals without colitis, phasic CRD elicited VMRs indicative of visceral pain, with the presence of acute mechanical sensitization; similar to that previously described in similar experimental conditions using the same distension paradigm.^46^ In animals with DSS‐induced colitis, similar responses to phasic CRD were observed. However, overall responses to CRD were increased when compared to those obtained in non colitic animals, thus suggesting the presence of inflammation‐associated hypersensitivity. Moreover, this response persisted over time, as assessed in repeated occasions between days 17 and 35 after the induction of inflammation. This suggests that the hyperalgesic responses associated to inflammation were long‐lasting, despite the resolution of colitis from a clinical point of view (see above). Indeed, we have observed a similar hyperalgesic state in animals with DSS‐induced colitis up to 49 days after the induction of inflammation (data not shown). Changes in VMRs during DSS‐induced colitis were, in magnitude, similar to those described in other models of inflammation‐associated sensitization, such as the TNBS‐induced colitis model,^13^, ^14^ but without the aggressiveness that characterizes the exposure to TNBS, as previously commented. Altogether, these observations suggest that DSS‐induced colitis results in a reproducible long‐term state of colonic mechanical hyperalgesia, despite the clinical colitis resolution, thus representing a feasible animal model to study this condition.

Visceral pain sensitization, either in humans or in relevant animal models, has been associated to peripheral and central sensitization mechanisms implying changes in sensory pathways leading to an increase in pain transmission and, therefore, to exacerbated pain manifestations (hypersensitivity).^54^, ^55^, ^56^ Inflammatory mediators, such as cytokines, released during inflammation have been directly implicated in peripheral sensitization. During DSS‐induced colitis, there is a significant local (colon) up‐regulation of pro‐inflammatory cytokines (present observations).^7^, ^26^, ^28^ This might contribute to the increased responses to CRD observed in animals with colitis. Moreover, an up‐regulation in pro‐inflammatory cytokines was also observed at central sites related with the processing of sensory signals arising from the colon (lumbosacral DRGs and lumbosacral region of the spinal cord).^57^, ^58^ Therefore, it is feasible to assume that a similar cytokine‐mediated sensitization process might take place at central (spinal) relevant areas, as previously shown in other models of intestinal inflammation.^56^ As a limitation of this study, it is worthy to consider that we have only evaluated expression changes at the mRNA, but not at the protein level, which might not necessarily follow the same pattern. Hence, we cannot discard that sustained increased plasma levels of pro‐inflammatory cytokines after clinical colitis resolution might contribute to the sensitization process. Altogether, these data suggest that a combination of peripheral and central (spinal) sensitization might occur during DDS‐induced colitis as the underlying mechanism explaining the mechanical hypersensitivity observed in this model. Interestingly, this reproduces the sensitization mechanisms postulated in humans with visceral (intestinal) hypersensitivity, such as during IBS, which is supposed to include central and peripheral alterations in pain processing.^4^, ^59^

To further understand the mechanisms mediating colitis‐associated visceral hypersensitivity, the peripheral (colon) and central (spinal cord) expression of different sensory‐related markers, mediating both pro‐ and anti‐nociceptive responses, was also assessed. Interestingly, differential tissue‐ (colon vs. spinal cord) and sensory‐related (pro‐nociceptive vs. anti‐nociceptive) modulatory responses were detected during inflammation. Within the colon, an up‐regulation of the pro‐nociceptive markers TRPV1 and Ramp1 was detected, thus suggesting a facilitation of nociceptive responses consistent with the state of hyperalgesia observed in the same animals. This agrees with the described role of TRPV1 and its modulation during inflammation in other animal models as well as in humans.^22^, ^60^, ^61^, ^62^ On the contrary, the up‐regulation of Ramp1 could lead to an increase of CGRP‐mediated pro‐nociceptive responses at peripheral sites.^63^ Within the lumbosacral spinal cord, an overall down‐regulation of pro‐nociceptive markers and an up‐regulation of anti‐nociceptive markers was observed. These responses might seem incongruent with the presence of hypersensitivity and central spinal sensitization (as discussed above), but, as a whole, they can be interpreted as the expression of compensatory mechanisms as an attempt to normalize nociceptive mechanisms and thus avoiding excessive/aberrant pain responses in states of sensitization. Here, we assessed the expression of a set of inflammatory mediators previously related to pain and sensitization mechanisms, but we cannot discard that other innate cytokines might be regulating these mechanisms. Alternatively, a non‐inflammatory regulation of nociceptors might also participate in the sensory changes observed here.

It is noteworthy that in animals exposed to DSS the pro‐nociceptive σ_1_R followed a reverse pattern of expression. It was down‐regulated at the periphery (colon) and up‐regulated at central levels (lumbosacral spinal cord). Activation of σ_1_Rs in the spinal cord produce mechanical hypersensitivity and increased pain responses in different animal models.^64^, ^65^ Therefore, the changes observed might support a direct role for σ_1_Rs on the development of inflammation‐associated central sensitization.

One of the distinctive characteristics of visceral pain is its capacity to generate referred somatic pain, revealed as sensitivity changes (hypersensitivity) that can be detected in the paws, tail, or even the abdominal wall.^5^, ^6^, ^7^ To explore this possibility, we also assessed the presence of referred somatic pain in animals with visceral hypersensitivity. For this, we evaluated time‐related mechanical sensitivity (withdrawal responses) of the hind paws in the same animals with DSS‐induced colitis used to assess viscerosensitivity. In the present conditions, moderate pro‐algesic responses to the mechanical probing of the hind paws were detected, without correlation with the visceromotor responses elicited by CRD, thus indicating a relatively poor association between DSS‐induced colitis and the induction of referred somatic hyperalgesia.

In summary, we present evidence supporting the feasibility of DSS‐induced colitis in rats as an experimental model of long‐lasting visceral hypersensitivity, reminiscent of the hypersensitive state observed in humans with functional and inflammatory GI disorders. The changes in viscerosensitivity observed are likely to result from the interaction between central (spinal cord) and peripheral (colon) sensitizing mechanisms implicating a site‐specific modulation of both pro‐ and anti‐nociceptive pathways and resulting in a persistent state of sensitization. The overall characteristics of the model make feasible its use in the characterization of new therapeutic targets and the validation of new drugs for the treatment of visceral pain originating within the GI tract. In any case, these results warrant further validation studies as to ensure the translational relevance of the model.

## AUTHOR CONTRIBUTIONS

VM, Conceptualization, Methodology, Investigation, Resources, Writing‐review & editing, Supervision. SL‐E, Methodology, Investigation, Formal analysis, Writing‐original draft, Writing‐review & editing. JML‐T, Methodology. MP, Methodology.

## CONFLICT OF INTEREST

The authors declare no competing interests.

## Supporting information


Figure S1
