# Supplementary material for: Long‐lasting visceral hypersensitivity in a model of DSS‐induced colitis in rats
Source: Neurogastroenterol Motil. 2022 Aug 5;34(11):e14441. doi: 10.1111/nmo.14441 (PMC9787759; doi:10.1111/nmo.14441)
Supplement: Supplementary file 1 — Figure S1 [file NMO-34-e14441-s001.docx]

**Long-lasting visceral hypersensitivity in a model of DSS-induced colitis in rats**

Sergio López-Estévez, Josep Manuel López-Torrellardona, Marc Parera, Vicente Martínez

Supplementary Fig. 1:

Correlation between visceromotor responses to CRD (AUC) and somatic pain responses (as assessed in the hind paws, relative to experimental day -1 (d-1), taken as basal sensitivity, 0% change). Visceral and somatic sensitivity were assessed in the same animals on consecutive days (see Fig. 1 for details of the experimental protocol). Each point represents an individual animal.
